# Optical Calibration of a Submicrometer Magnification Standard

**DOI:** 10.6028/jres.097.008

**Published:** 1992

**Authors:** Jon Geist, Barbara Belzer, Mary Lou Miller, Peter Roitman

**Affiliations:** National Institute of Standards and Technology, Gaithersburg, MD 20899

**Keywords:** calibration, electron microscope, magnification standard, scanning electron microscope, transmission electron microscope

## Abstract

The calibration of a new submicrometer magnification standard for electron microscopes is described. The new standard is based on the width of a thin thermal-oxide film sandwiched between a silicon single-crystal substrate and a polysilicon capping layer. The calibration is based on an ellipsometric measurement of the oxide thickness before the polysilicon layer is deposited on the oxide. The uncertainty in the derivation of a thickness for the layer from the ellipsometric parameters is also derived.

## 1. Introduction

It was recently proposed [1] that the same calibration technique [2] used to produce the NIST 2530 Standard Reference Material (SRM) series of oxide thickness standards could be used as the basis for a new submicrometer magnification standard. The key ideas for the new standard are the following: 1) A thin thermal oxide is grown on a 76.2 mm (3 in diameter) silicon wafer, 2) the thickness of the oxide is measured at the center of the wafer with the NIST High-Accuracy Ellipsometer [3], 3) the variation in oxide thickness is measured at nine points over the wafer using a high-precision reflectometer, and 4) a polysilicon cap is deposited over the oxide layer subsequent to certification of the oxide thickness. Sections of the resulting wafer can be used as submicrometer magnification standards when viewed edge on. For use in a Scanning Electron Microscope (SEM), the cleaved edge must be dipped in a weak solution of HF for a few seconds before viewing in order to create topographic features. For use in a Transmission Electron Microscope (TEM), the standard can be sectioned in the usual way.

This paper describes the calibration of this new standard and the associated uncertainty analysis. There are two very different types of uncertainty associated with this standard. The first is the uncertainty in the thickness of the oxide at any point on the wafer. This uncertainty, which is reported in the calibration certificate, is described here. The second is the uncertainty associated with the resolution of the oxide-silicon interfaces in an electron microscope. This uncertainty will vary depending upon the machine and the operating conditions, and must be estimated by the user. With some SEMs, this uncertainty will be much larger than the calibration uncertainty, and with some TEMs, it may be smaller than the calibration uncertainty. In either case, these two uncertainties are added in quadrature because they are uncorrelated. This paper is concerned only with the calibration of the oxide thickness, and does not address the uncertainty associated with the actual use of the standard in an electron microscope.

**Fig. 1:**
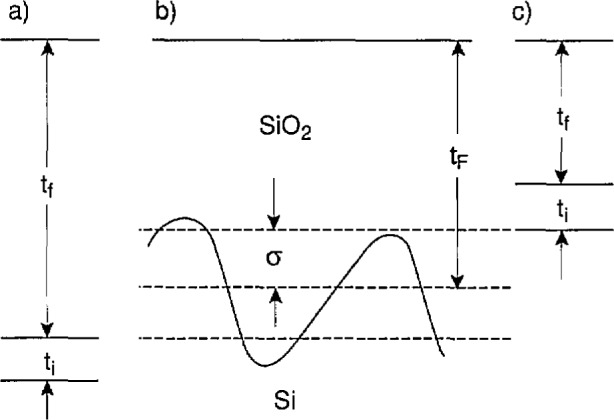
Optical model of a thermal oxide on a single-crystal silicon substrate that was used to derive an oxide thickness from measurements of ellipsometric *Δ* and *ψ* data for the principal angle at 633 nm.

A model of the optical properties of the magnification standard is needed to extract the thickness of the oxide layer from the measured ellipsometric parameters. The model used for the silicon/silicon dioxide system consists of three layers as shown in Fig. 1: 1) a homogeneous, isotropic top layer characterized by a film thickness *t*_f_ and a real index of refraction *n*_f_; 2) a homogeneous, isotropic interlayer characterized by an interlayer thickness *t*_i_, and a real index of refraction *n*_i_; and 3) a homogeneous, isotropic substrate characterized by a complex index of refraction *n*_s_. The three-layer model is used because Taft and Cordes showed that a two-layer model produces an oxide index of refraction that depends upon oxide thickness, which is clearly unphysical [4]. The bottom layer is interpreted as single-crystal silicon and the top layer as amorphous silicon-dioxide. The interpretation of the interlayer in terms of a physical structure is less straightforward, and is discussed in detail later in this paper.

It is not possible to determine *t*_f_, *t*_i_, *n*_f_, *n*_i_, and *n*_s_ from measurements of the ellipsometric parameters [2] *Δ* and *ψ* on a single wafer. The derivation of the values reported in the certificate of calibration for this new standard is carried out on a lot of wafers as described in Ref. [2]. The remainder of this paper describes in more detail how a lot of wafers is measured, how the oxide thickness is calculated, and how the uncertainty in the oxide thickness is estimated. The details provided here complement those presented in Ref. [2], with the exception that *t*_f_*+t*_i_ is used as the oxide thickness in Ref. [2], whereas *t*_f_*+t*_i_/2 is used for the oxide thickness for this magnification standard. The main reason for this change is that the ellipsometric standard is concerned with the thicknesses of layers defined by an optical model, whereas this standard is concerned with the thickness of a physical layer, and *t*_i_/2 is more consistent with alternative physical models of the optically determined interlayer. This point is explained in more detail later in this paper.

## 2. Definition of a Batch and a Lot of Wafers

**Fig. 2:**
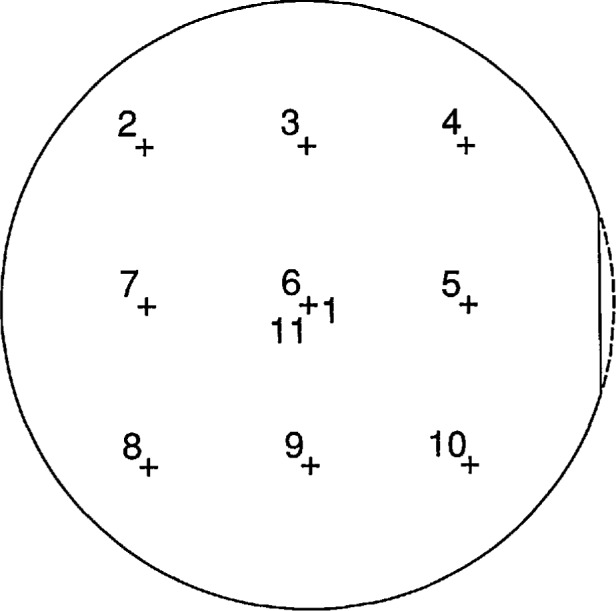
Order in which the thickness of the oxide is measured relative to the center of the wafer at the points denoted by crosses. The crosses are on 2 cm centers. The center is measured as points 1, 6, and 11.

For the purposes of this magnification standard, a batch of wafers is defined as a set of at least five wafers that were put into an oxide-growth furnace at the same time, so all of the wafers in the batch have the same growth conditions and same nominal oxide thickness. A batch of wafers is processed and measured in the following steps:

1. The wafers are cleaned and dried immediately before loading into an oxide-growth furnace.
2. A nominal thickness oxide is grown on the wafers.
3. The relative variation of the oxide thickness is measured at nine points on the surface of each wafer as shown in Fig. 2.
4. A simple model of the variation in oxide thickness with position on the wafer is fit to the data measured in Step 3 above, as discussed later in this paper.
5. The ellipsometric parameters Δ and ψ at the center of each wafer for the principal angle of incidence are measured at 633 nm with the NIST High-Accuracy Ellipsometer [3].
6. A layer of polysilicon is deposited over the oxide.

A lot of wafers is defined to be a set of at least three batches of wafers. To qualify as a lot, 1) at least one batch must consist of wafers with nominal 50 nm oxides, another with nominal 100 nm oxides, and a third with nominal 200 nm oxides, and 2) all oxides must have been grown at the same temperature with the same gas-flow conditions in the same furnace. Batches of wafers with nominal 12 and 25 nm oxides may also be included in a lot on a fairly routine basis, and batches of other thicknesses may be included on occasion. The thicknesses of 50, 100, and 200 nm were chosen for consistency with Ref. [2]; the restrictions on lots and batches of wafers are required because *n*_f_ and either *n*_i_, or *t*_i_ have been determined to depend upon growth conditions [4].

When all of the wafers in all of the batches constituting a lot have been measured, the thicknesses *t*_f_ of the oxide films on the individual wafers in the lot are adjusted in a least-squares fit [2,5] to the experimental *Δ* and *ψ* data based on the model shown in Fig. 1. The extinction coefficient *k*_s_ of the silicon substrate at 633 nm is assumed to be the same for all of the wafers in the lot, and is fixed at 0.0156 ±0.0003 [6]. The indices of refraction *n*_s_, *n*_f_, and *n*_i_, of the silicon substrate, the silicon-dioxide film, and the interlayer, respectively, are assumed to be the same for all wafers in the lot, and are adjusted to produce the best fit. The thickness *t*_i_ of the interlayers is also assumed to be the same for all wafers, and is also adjusted in the fit [4].

## 3. Oxide Thickness and Uncertainty

The thickness of the oxide at any point (*x,y*) on the wafer for which −2 cm ⩽ × ⩽ 2 cm, and −2 cm ⩽ y ⩽ 2 cm can be calculated from the values of *t*_f_, *t*_i_, α, and β reported on the certificate of calibration for the new magnification standard by using

$$t\left(x,y\right)=t_{f}+t_{i}/2+\alpha x+\beta y.$$ (1)

The point (0,0) is the center of the circular extension of the circumference of the wafer as shown in Fig. 2. Equation (1) assigns *t*_f_*+t*_i_/2 as the oxide thickness at the center of the wafer. The reason for this choice is associated with the interpretation of the interlayer, and is described in the sections of this paper devoted to that topic.

The estimated standard deviation of *t(x,y)* is given by

$$\Delta t={\left(\Delta {t_{f}}^{2}+\Delta {t_{i}}^{2}+{t_{i}}^{2}+{t_{i}}^{2}+\Delta {t_{\text{map}}}^{2}+\Delta {t_{\text{poly}}}^{2}\right)}^{1/2}.$$ (2)

The quantity *Δt* is the uncertainty in the oxide thickness, and it is reported in the calibration certificate. The uncertainties in *α* and *β* are so small that they do not contribute significantly to the overall uncertainty, and they are not reported. The nature of the uncertainties contributing to *Δt* and how they were calculated is described below.

The quantities *Δt*_f_ and *Δt*_i_ in Eq. (2) are the uncertainties associated with the adjustment of *t*_f_ and *t*_i_ in the fit referred to in the previous section. In principle, *Δt*_f_ and *Δt*_i_ vary from wafer to wafer, but in fact they are dominated by systematic errors that are common to all of the ellipsometric measurements, so *Δt*_f_ and *Δt*_i_ are the same for all wafers, even from different lots and batches. The first *t*_i_ in Eq. (2) is an uncertainty associated with the use of the model of Fig. 1. The second *t*_i_ is an uncertainty that accounts for any microscopic roughness of the oxide-silicon interface and any microscopic variations in the oxide thickness. Microscopes are sensitive to these variations, but the High-Accuracy Ellipsometer is not, due to its macroscopic beam size, which is measured in millimeters. The uncertainty *Δt*_map_ is the residual standard deviation of the fit of the model of Eq. (1) to the measured data on the variation of oxide thickness over the surface of the wafer, and *Δt*_poly_ is an uncertainty associated with assigning a thickness to the oxide under the polysilicon on the basis of measurements carried out before the polysilicon was deposited.

## 4. Film and Interlayer Thickness

The uncertainties *Δt*_f_ and *Δt*_i_ are determined as described in Ref. [2]. They are one-sigma estimates for the sum in quadrature of the uncertainties associated with both the random errors and the systematic errors in the measured values of *Δ* and *ψ* for the individual wafers in the lot under the assumption that the model in Fig. 1 accurately describes the wafers. The problem with this assumption is that different interpretations of the interlayer require that it be apportioned differently between the top oxide layer and the silicon substrate.

## 5. Interpretation of Interlayer

The physical interpretation of the interlayer shown in Fig. 1 is not straightforward because different experimental results reported in the literature do not appear to be consistent. The interlayer has been interpreted as a graded layer of SiO*_x_* where the *x* value varies from 0 on the silicon side of the Interlayer to two on the oxide side of the interlayer [4]. This interpretation is consistent with the fact that the optically derived thickness for the interlayer varies from 0.5 to 2.0 nm, depending upon the particular samples constituting a lot of wafers. This interpretation is also supported by x-ray photoemission spectroscopy (XPS) measurements that detect silicon in every allowed oxidation state at the interface [7]. According to this interpretation, most of the interlayer (if not all of it) should be considered part of the oxide.

On the other hand, TEM [8] and grazing incidence x-ray scattering measurements [9] on thermal oxides grown between room temperature and 900 °C on (100) silicon surfaces detect only a single atomic layer of silicon that is not bonded either like single-crystal silicon or like silicon dioxide. This result is not necessarily inconsistent with the XPS results. Some of the dangling bonds associated with the specially bonded layer of silicon atoms could be dimerized or tied up by bridging oxygen atoms to create the various XPS peaks associated with the remaining oxidation states. (It does not seem possible to determine the concentrations of different species from the heights of the XPS peaks.)

Even though the TEM and grazing-incidence x-ray scattering results are not necessarily inconsistent with the XPS results, the former strongly suggest that the interlayer should be 0.5 nm thick, and should not vary from lot to lot. These results also predict that the interlayer should be considered part of the silicon in some experiments such as TEM measurements, but part of the oxide in other experiments such as those in which etches remove the atypically bonded atomic layer of silicon atoms as well as the oxide.

**Fig. 3:**
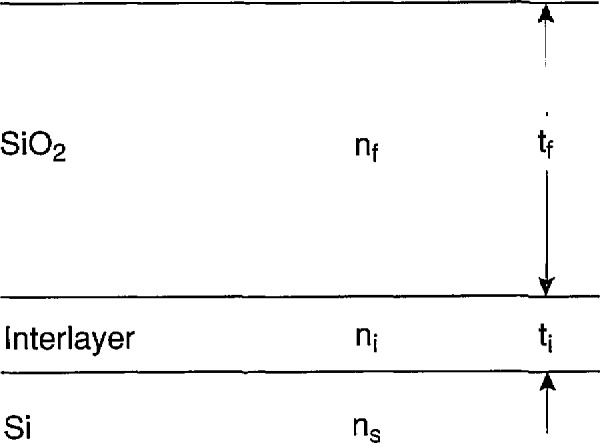
Schematic illustration of plausible worst-case limits a) and c) between the film and interlayer thicknesses *t*_f_ and *t*_i_, in the optical model and the average oxide thickness *t*_F_ if the interlayer is an artifact that describes the change in polarization induced by the roughness at the oxide-silicon interface as shown in b). If this is the case, *t*_f_ and *t*_i_ are adjusted to produce the same polarization of the radiation leaving the oxide surface as is produced by the roughness and *t*_F_, and *t*_F_ need not equal *t*_f_.

A possible resolution of this paradox is that some portion of the optically derived width of the interlayer is an artifact that produces a better fit to the experimental data because it involves an extra free parameter, but does not correspond exactly to any real structure at the interface. For example, the interlayer in the optical model might be compensating for the polarization properties introduced by the roughness of the silicon surface and the microscopic variations in thickness of the oxide layer [10]. This situation is illustrated in Fig. 3.

## 6. Microscale Roughness

Figure 3b represents a rough interface between the oxide and the silicon substrate for spatial frequencies great enough so that the top surface of the oxide is not conformal with the interface under the assumption that such spatial frequencies exist. The average oxide thickness is denoted by *t*_F_ and the root-mean-square (rms) roughness is denoted by *σ*, and it is assumed that the ellipsometric parameters *Δ*(*σ*) and *ψ*(*σ*) depend upon *σ.* Therefore, *t*_f_ and *t*_i_, which are obtained by fitting the optical model to the measured *Δ* and *ψ* data for a wafer, also vary with *σ*. In fact, there is no reason that *t*_f_ should be identical to *t*_F_ even though this result would be intuitively satisfying.

The only way to definitively determine the dependence of *t*_f_ and *t*_i_ on *σ* is to solve Maxwell's equations for oblique incidence on a rough surface. This is not within the scope of this paper. However, there is some evidence that *t*_i_ ≃ *σ for σ* < 5 nm [11], and Figs. 3a and 3c shows worst-case estimates for the limits of variation of *t*_f_ with *σ.* For one limit, the average film thickness is given by

$$T_{F}+t_{f}+t_{i}+\sigma ,$$ (3)

and for the other limit it is given by

$$t_{F}=t_{f}-\sigma .$$ (4)

This range is covered by

$$t_{F}=t_{f}+t_{i}/2\pm \left(t_{i}/2+\sigma \right).$$ (5)

This is the justification for the first two terms in Eq. (1).

The range ± (*t*_i_/2 *+ σ*) represents a limit of error. What is needed for Eq. (2) is something more like a one-standard deviation estimate. This can be obtained by multiplying the range by 2/3. If we now use *t*_i_ as an approximation to *σ* as suggested in Ref. [11], the uncertainty becomes *±t*_i_. This is the first *t*_i_ appearing in Eq. (2).

The uncertainty just described accounts only for the model uncertainty. An additional ± *σ* must be added to allow for the possibility that the microscope might sample the oxide thickness at a crest, a trough, or or any other part of the micro-rough oxide-silicon interface illustrated in Fig. 3. Once again, we approximate *σ* by *t*_i_, and obtain the second *t*_i_ appearing in Eq. (2). Interpretation of the entire interlayer as an artifact of roughness as shown in Fig. 3 is a worst-case scenario. The other interpretations of the interlayer that were discussed above are all described by Eq. (5), and the stated uncertainties should be sufficiently conservative.

## 7. Variations in Oxide Thickness

Before the polysilicon is deposited on the wafer, the thickness *t*_n_ of the oxide on each wafer is measured with a reflectometer for the points *n* = 1, 2, 3, …, 11 indicated in Fig. 2. The parameters *α*, *β*, and *t* in the equation

$$t_{n}=t+\alpha x_{n}+\beta y_{n}$$ (6)

are adjusted in a least-squares fit to the measured data points, where *x_n_* and *y_n_* are given in Table 1. The parameters *α* and *β* describe the variation of oxide thickness about the thickness at the center of the wafer, and are reported in the calibration certificate. The residual standard deviation of the fit is used as *Δt*_map_ in the calculation of the uncertainty in Eq. (2), and if any single residual is larger than 0.6 nm, the wafer is rejected for use as a standard. The value *t* is not reported, since *t*_f_ *+ t*_i_/2, which is derived from the measurements made with the High-Accuracy Ellipsometer, is a more accurate estimate of the thickness of the oxide at the center of the wafer.

The symmetric location of the points in Fig. 2 about the wafer center greatly simplifies the least-squares analysis [12], so

$$\alpha =\frac{\left(\sum x_{n}t_{n}\right)}{24{\text{mm}}^{2}},$$ (7)

$$\beta =\frac{\left(\sum y_{n}t_{n}\right)}{24{\text{mm}}^{2}},$$ (8)

and

$$\Delta =\Delta t_{\text{map}}/9.4\text{mm},$$

where *Δ* is the uncertainty in the coefficients *α* and *β*.

## 8. Thickness of the Oxide Under the Polysilicon

The following experiment was conducted to set an upper limit on the change in thickness of the oxide caused by deposition of the polysilicon layer. Seven wafers with oxides with nominal 100 nm oxides were selected, and the oxide thicknesses were measured at the center of each wafer with the High-Accuracy Ellipsometer. A layer of polysilicon similar to that used on the new submicrometer magnification standard was deposited over the oxide on each wafer, and was removed after the wafer cooled to room temperature by etching in 14 *M* KOH for 30 min at room temperature [13]. The wafers were then rinsed for 15 min in deionized H_2_O at room temperature. The thicknesses of the oxides were then remeasured. The average value of the difference between the oxide thickness before and after the deposition and etch was 0.6 nm with a standard deviation of the mean of seven measurements of 0.1 nm. The difference is used for *Δt*_poly_ in Eq. (2). This is a conservative estimate because the decrease in thickness of the oxide layer is more likely associated with the removal of the polysilicon than with its deposition.

## 9. Conclusion

Typical values of *Δt* and of the terms in Eq, (2) that contribute to it are listed in Table 2. It is clear that the uncertainties of magnitude *t*_i_ completely dominate the other uncertainties listed in Table 2 in their contribution to *Δt* through addition in quadrature. As far as this standard is concerned, there is no reason to attempt to decrease any of the other uncertainties listed in Eq. (2) until the physical meaning of the interlayer derived from the optical model is clearly understood, so that these uncertainties can be replaced by smaller values. Nevertheless, the uncertainty *Δt* in the calibration of the oxide thickness is quite satisfactory for many applications, and will, in fact, be much smaller than the errors associated with the use of the standard in many electron microscopes.

## Figures and Tables

**Table 1:** Relation of position coordinates *x* and *y* to measurement number in uniformity map of oxide thickness

Measurement No.	*x* (cm)	*y* (cm)
1	0.0	0.0
2	−2.0	2.0
3	0.0	2.0
4	2.0	2.0
5	2.0	0.0
6	0.0	0.0
7	−2.0	0.0
8	−2.0	−2.0
9	0.0	−2.0
10	2.0	−2.0
11	0.0	0.0

**Table 2:** Typical values for the uncertainties in the oxide thick ness

Error term	Value (nm)	Source of error
*Δt*_f_	0.5	*t*_f_ from fit
*Δt*_i_	*0.4*	*t*_i_ from fit
*t*_i_	1.4	meaning of interlayer
*t*_1_	1.4	possible roughness of interface
*Δt*_map_	0.3	variation in oxide thickness
*Δt*_poly_	0.6	deposition of polysilicon
*Δt*	2.2	sum in quadrature
