# Erratum: Optical Calibration of a Submicrometer Magnification Standard

**DOI:** 10.6028/jres.097.027

**Published:** 1992

**Authors:** Jon Geist, Barbara Belzer, Mary Lou Miller, Peter Roitman

**Affiliations:** National Institute of Standards and Technology, Gaithersburg, MD 20899

[J. Res. Natl. Inst. Stand. Technol. Volume 97, Number 2, March–April 1992, 267]

The figures over figure captions 1 and 3 should be interchanged.

